# Exercise-Mediated Molecular Mechanisms of Antidepressant Effects: The Role of the Microbiota–Gut–Brain Axis

**DOI:** 10.3390/ijms27167291

**Published:** 2026-08-15

**Authors:** Xin Kuang, Xinyan Zheng

**Affiliations:** School of Exercise and Health, Shanghai University of Sport, Yangpu, Shanghai 200438, China; 2421518038@sus.edu.cn

**Keywords:** depression, gut, microbiota, dysbiosis, exercise, brain

## Abstract

Depression is a prevalent and complex neuropsychiatric disorder that substantially impairs quality of life worldwide. Current pharmacological and psychotherapeutic interventions are often limited by inadequate efficacy and restricted accessibility. Exercise has emerged as a potent non-pharmacological alternative, exhibiting antidepressant effects comparable to conventional treatments, with the microbiota–gut–brain (MGB) axis increasingly recognized as a key mediator. Despite growing interest, existing research articles largely describe associations without mapping the molecular pathways from exercise-altered microbes to neurobiological changes in depression. This review addresses these gaps by comprehensively dissecting the molecular cascades from microbial remodeling to synaptic and systemic adaptations, thereby providing a mechanistic roadmap absent in current syntheses. We first delineate how gut dysbiosis contributes to depression via endocrine, neural, and immune pathways. Subsequently, we highlight the ability of exercise to reverse these pathological states by restoring microbial diversity, reinforcing intestinal barrier function, and modulating the production of microbial metabolites. These exercise-driven changes collectively attenuate systemic and neuroinflammation, enhance brain-derived neurotrophic factor signaling, augment serotonin synthesis, and dampen hypothalamic–pituitary–adrenal axis hyperactivity. By reinstating intestinal homeostasis, exercise acts as a central regulator of the MGB axis and represents a promising therapeutic strategy. This integrative framework offers novel insights into depression pathogenesis and opens avenues for multifaceted intervention.

## 1. Introduction

Depression is a multifaceted neuropsychiatric disorder characterized by persistent anhedonia, fatigue, and sleep disturbances, which profoundly impact cognitive function, emotional stability, and physical well-being [1]. In recent years, the global prevalence of depression has risen significantly, notably Major Depressive Disorder (MDD), which is recognized as a severe and highly disabling condition. Data from the Global Health Data Exchange indicate that over 300 million individuals worldwide are currently affected [2]. The high prevalence of this chronic disease not only diminishes quality of life but also imposes a substantial socioeconomic burden [2]. Consequently, it is imperative to elucidate the underlying pathophysiological mechanisms to develop more effective therapeutic strategies.

While the precise pathogenesis of depression disorders remains incompletely elucidated, emerging evidence implicates the microbiota–gut–brain (MGB) axis as a potentially significant mechanistic pathway [3]. As a central mediator of this axis, the gut microbiota consists of diverse bacterial communities and their metabolites residing in the gastrointestinal tract. These microbial elements bidirectionally regulate brain–gut interactions via the MGB axis through neural, immune, endocrine, and metabolic signaling routes [4]. This bidirectional signaling is essential for modulating stress responses, cognitive function, and the development of neuropsychiatric conditions [5,6]. Accumulating evidence suggests that gut microbiota dysbiosis compromises intestinal barrier integrity and perturbs microbial homeostasis. These pathological alterations subsequently dysregulate key signaling cascades, including the hypothalamic–pituitary–adrenal (HPA) axis, vagus nerve (VN) afferent signaling, and neuroinflammatory processes [7], all of which are functionally linked to depression [8]. Together, these findings underscore the pivotal role of the gut microbiota in the development of depressive disorders.

Currently, first-line treatments for depression include psychotherapy, pharmacotherapy, or their combination. However, these options are often constrained by the side effects of antidepressants [9] and the high cost or limited accessibility of psychotherapy [10]. Exercise has emerged as a promising non-pharmacological intervention, with demonstrated efficacy in alleviating depressive symptoms and reducing MDD risk [11]. Notably, exercise may offer comparable or even superior antidepressant benefits relative to other treatment modalities [12]. Beyond its direct mood-regulating effects, exercise profoundly influences the structure and function of the gut ecosystem. Long-term physical exercise has been shown to enhance α-diversity of the gut microbiota, promote colonization by recognized beneficial genera such as Bifidobacterium, and suppress the overgrowth of opportunistic pathogens [13]. Further analyses reveal that exercise increases the Firmicutes-to-Bacteroidetes ratio and enriches butyrate-producing taxa, augmenting short-chain fatty acid (SCFA) production [14,15]. However, the precise mechanisms underlying this effect remain to be elucidated.

These exercise-induced gut microbial alterations offer a novel perspective for understanding the antidepressant effects of physical activity. Patients with depression frequently exhibit a distinct gut dysbiosis profile, characterized by increased abundances of Bacteroidetes, Proteobacteria, and Actinobacteria alongside decreased Firmicutes [16]. Exercise has been shown to restore microbial homeostasis [17], and these microbial changes may be associated with multiple antidepressant mechanisms, including the reduction in neuroinflammation, the regulation of neurotransmitter metabolism, and the modulation of HPA axis function. Preliminary studies have further suggested that exercise-induced alterations in gut microbial diversity are associated with improvements in depressive-like behaviors [18]. Collectively, the modulatory effects of exercise on the gut microbiota may constitute an important initiating mechanism for its antidepressant actions, although the causal links within this complex network require further elucidation.

Despite these promising findings, critical knowledge gaps remain that warrant this review. While existing evidence has linked exercise to both gut microbial shifts and antidepressant effects, these observations remain largely fragmented, with limited efforts to integrate the endocrine, neural, and immune signaling cascades mediating the gut–brain dialogue into a coherent framework. Moreover, most available studies have primarily reported descriptive and correlative changes in gut microbiota accompanying exercise, without establishing whether these microbial shifts play a causal or mediating role in the antidepressant response. To address these gaps, our review aims to elucidate how gut dysbiosis contributes to depressive pathophysiology via endocrine, neural, and immune pathways, to examine how exercise restores microbial homeostasis and triggers signaling along these pathways, and to develop an integrative analytical framework that links specific microbial taxa and metabolites to distinct neurobiological targets. By synthesizing the available evidence, this review provides a guiding framework for future mechanistic investigations and the development of precision exercise-based interventions for depression. Nevertheless, several key questions remain unresolved, including how to optimize exercise modalities and dosages for maximal antidepressant efficacy, the temporal dynamics of microbial changes during exercise intervention, and the applicability of current evidence to heterogeneous patient subgroups.

## 2. Methods

This article was conducted as a narrative mechanistic review. Relevant publications were identified through searches of PubMed, Web of Science, and Google Scholar for studies published between 2010 and July 2026, with the final search conducted in July 2026. Search terms combined concepts related to exercise or physical activity, the gut microbiota or microbiota–gut–brain axis, depression or major depressive disorder, and candidate molecular pathways, including the HPA axis, vagus nerve, neuroinflammation, BDNF, serotonin, tryptophan metabolism, short-chain fatty acids, and endocannabinoid signaling. The reference lists of relevant articles were also examined to identify additional publications.

The review considered peer-reviewed English-language publications relevant to the relationships among exercise, the gut microbiota, and depression. Original human studies and preclinical animal studies were included to provide clinical and mechanistic perspectives, respectively. Human studies involving depressive disorders of different types and severities were considered, whereas animal studies were included when they assessed depressive-like phenotypes or related molecular mechanisms. Exercise modalities included aerobic, resistance, and combined training. Selected review articles were also consulted to provide conceptual background and identify additional relevant evidence. Case reports, letters to the editor, conference abstracts, and non-peer-reviewed publications were not considered.

Given the narrative and mechanistic scope of this review, the literature search was intended to identify representative and mechanistically informative evidence rather than to exhaustively capture all eligible studies. Findings were synthesized qualitatively according to the principal pathways discussed in the review. Preclinical studies were used primarily to evaluate mechanistic plausibility, whereas human observational and intervention studies were considered separately to assess clinical associations and translational relevance. A formal risk-of-bias assessment was not undertaken.

## 3. Gut Microbiota and Depression

### 3.1. Gut Microbiota Dysbiosis and Depression

The gut microbiota represents the most expansive and intricate microbial ecosystem within the mammalian digestive tract, comprising bacteria, fungi, viruses, and archaea, among which bacteria predominate [19]. This complex community is essential for maintaining systemic and intestinal homeostasis. Under physiological conditions, the gut microbiota exists in a state of dynamic equilibrium. However, this balance is highly sensitive to perturbations from diverse intrinsic and extrinsic factors such as genetics, diet, and physical activity, which can collectively precipitate a transition toward microbial dysbiosis [20]. Subsequently, such pathological shifts in microbial composition can profoundly impact neurological health and contribute to the pathogenesis of neuropsychiatric disorders, most notably depression. Preclinical studies have identified microbial alterations in rodent models exhibiting depressive-like phenotypes. For example, one mouse study reported reduced abundances of Lactobacillaceae, Prevotellaceae, Ruminococcaceae, and Lachnospiraceae, together with enrichment of Muribaculaceae and Bacteroidaceae [21]. These findings provide evidence of model-specific microbial alterations associated with depressive-like states. However, because rodents and humans differ substantially in gut microbial composition, physiology, diet, and experimental exposure, taxonomic patterns observed in rodent models should not be assumed to occur identically in patients with depression.

Human cohort studies should therefore be considered separately from these preclinical observations. Clinical studies have reported differences in microbial diversity and the relative abundance of specific taxa between individuals with depressive disorders and healthy controls [6,22,23,24]. These changes are observed across multiple taxonomic levels. At the phylum level, a characteristic increase in Bacteroidetes and Proteobacteria is often observed, alongside a concomitant reduction in Firmicutes [16]. Consistent with these observations, a reduced Firmicutes-to-Bacteroidetes (F/B) ratio has been reported in some cohorts [25]. However, the direction of F/B ratio changes is not consistent across studies. Although some studies have reported a lower F/B ratio in MDD, others have found an elevated ratio in particular populations or clinical subgroups. For instance, an early study by Jiang et al. [16] reported decreased Bacteroidetes and increased Firmicutes in MDD patients, resulting in an elevated F/B ratio. Furthermore, the association between F/B ratio changes and depression severity appears to vary across studies, with some reporting a higher F/B ratio in patients with severe depression compared to those with mild depression [26].

At the family level, the abundance of Providentiaceae, particularly within the genus Providencia, is significantly elevated, whereas genera harboring putative probiotic properties, such as Faecalibacterium and Ruminococcus, are markedly depleted [3,27]. Similarly, patients with depression exhibit a decline in psychotropic-related beneficial bacteria, such as Lactobacillus and Bifidobacterium [28]. At the genus and species levels, several opportunistic or inflammation-associated taxa, including Morganella, *Klebsiella pneumoniae*, and *Escherichia coli*, have been reported at higher relative abundance in some cohorts with depressive symptoms [29,30]. In a study by Zhao et al. [31], a marked reduction in the abundance of *Bifidobacterium longum* in individuals diagnosed with MDD, a reduction that may adversely affect neural function and may be associated with depressive pathology. In a large-scale study of nearly 2600 participants with depressive symptoms, Radjabzadeh et al. [29] identified a significant correlation between depressive symptoms and an increased abundance of Morganella and *K. pneumoniae*. However, these findings are associative and should not be interpreted as evidence that these taxa are uniformly pathogenic across all strains, hosts, or ecological contexts. In particular, the association between *Morganella* and depressive symptoms has been hypothesized to involve systemic immune activation, but direct causality has not been established [29]. At a broader taxonomic level, a meta-analysis [32] reported that patients with MDD exhibit a marked depletion of bacterial families such as Veillonellaceae, Prevotellaceae, and Sutterellaceae, alongside a reduction in the genera Coprococcus, Faecalibacterium, Ruminococcus, and Bifidobacterium. Higher abundances of Actinobacteriaceae and Paraprevotella were also reported. Nevertheless, the direction and magnitude of these differences vary considerably across studies, suggesting that depression is unlikely to be characterized by a single universal taxonomic profile. Altogether, gut microbial diversity is not static but rather in a state of continuous flux, shaped by multiple variables, including host physiological states, environmental factors, and lifestyle habits. Given the high degree of inter-individual variability and temporal dynamics in microbial composition, further investigation is warranted to elucidate its changing patterns and clinical implications.

### 3.2. Gut Microbiota Metabolites and Depression

The symbiotic interaction between the gut microbiota and the host is orchestrated by a sophisticated network of bioactive signaling molecules, which are essential for maintaining both gastrointestinal homeostasis and neurological health. These mediators primarily encompass fermentation-derived metabolites, host-derived compounds modified by microbial enzymes (particularly bile acids), and direct microbial secretory products [33]. Among microbial metabolites, SCFAs, including acetate, propionate, and butyrate, are considered the primary functional outputs of microbial fermentation with potent biological activity [34]. Previous evidence has indicated that patients with MDD exhibit lower fecal SCFA concentrations compared to healthy individuals [35]. This finding suggests that SCFA deficiency may be intimately linked to the pathogenesis of depressive disorders. Specifically, the physiological significance of SCFAs extends beyond their role as energy sources for colonocytes; they exert pleiotropic effects through the stimulation of G protein-coupled receptors (GPCRs), such as GPR41 and GPR43, which are expressed in multiple tissues, including adipose tissue, distal ileum, colon, and immune cells, as well as through the inhibition of histone deacetylases (HDACs), thereby modulating gene transcription and metabolic pathways with systemic consequences [36,37]. In the context of depression, these diverse bioactivities translate into multiple protective mechanisms, including the maintenance of intestinal barrier integrity via tight junction protein regulation [38] and the modulation of systemic immune responses through macrophage activity [39].

Notably, the MDD group was found to have a significant decrease in the abundance of butyrate-producing bacteria such as Lachnospira, Subdoligranulum, Blautia, and Dialister [40]. Among SCFAs, butyrate is of particular clinical importance because it serves as the primary energy substrate for colonocytes and acts as a potent epigenetic modulator by inhibiting HDACs [41]. This inhibition facilitates chromatin remodeling, thereby influencing the transcription of genes associated with neuroprotection, synaptic plasticity, and inflammatory control. Additionally, butyrate functions as a ligand for peroxisome proliferator-activated receptor γ (PPARγ) and the aryl hydrocarbon receptor, both of which are implicated in the regulation of immune homeostasis and neuroinflammatory responses [42,43]. Accordingly, therapeutic restoration of SCFAs levels, especially through butyrate supplementation, has demonstrated considerable potential in alleviating depressive symptoms by multiple mechanisms, including reinforcement of intestinal barrier function, attenuation of HPA axis hyperactivity, and promotion of neuroplasticity [21,44].

In parallel, bile acids represent another crucial class of microbially modified metabolites. They engage in sophisticated cross-talk through the nuclear farnesoid X receptor (FXR) and the Takeda G-coupled protein receptor 5 (TGR5), thereby regulating neuroendocrine pathways and neurotransmitter homeostasis [45]. Depressive pathology is frequently associated with dysregulated bile acids profiles, often driven by the loss of taxa essential for bile acids biotransformation, such as Turicibacter spp. [22]. Additionally, emerging evidence suggests that other microbial products, including lactate and B-group vitamins, contribute to depression via pathways involving systemic immune activation, mitochondrial energy metabolism, and the synthesis of neurotransmitter precursors [46,47]. Accumulating evidence thus underscores that both the structural composition of the gut microbiota and its dynamic metabolic fluctuations serve as indispensable determinants in the multifactorial pathogenesis of depression, highlighting the therapeutic potential of targeting microbial metabolic pathways as a novel antidepressant strategy.

### 3.3. The Microbiota–Gut–Brain Axis and Depression

The gut microbiota communicates with the brain through the MGB axis, a sophisticated, multi-layered biological system that integrates neural, immune, endocrine, and metabolic signaling to regulate cognitive function and emotional states [48]. This complex axis encompasses several key components, such as immune mediators including cytokines that form a major part of the immune pathway, alongside the VN serving as the primary neural conduit, the HPA axis as the central neuroendocrine regulator, and critical neurotransmitters like serotonin (5-hydroxytryptamine, 5-HT) [49,50]. A growing body of evidence demonstrates that chronic stress can disrupt the balance of the gut microbiota, thereby activating the systemic immune system. This process further triggers inflammatory mechanisms in the brain, ultimately contributing to the development of depression [51]. Specifically, in depressive disorders, microbial and metabolic shifts perturb intestinal homeostasis and compromise barrier integrity. This facilitates the production and translocation of bacterial endotoxins, notably lipopolysaccharide (LPS) from Gram-negative bacteria. LPS subsequently activates immune cells through the Toll-like receptor 4 (TLR4) signaling pathway, stimulating the release of pro-inflammatory cytokines, including interleukin-1β (IL-1β), interleukin-6 (IL-6), and tumor necrosis factor-alpha (TNF-α), and triggering a local intestinal inflammatory response [52]. These inflammatory mediators then propagate systemically to degrade tight junction proteins at the blood–brain barrier (BBB) in regions essential for emotional processing, such as the prefrontal cortex, hippocampus, and striatum. The resulting increase in BBB permeability permits cytokine infiltration into the central nervous system (CNS), where they induce neuroinflammation, neuronal damage, and impaired neurogenesis, ultimately manifesting as depressive symptoms [53].

Within neural pathways, the VN serves as the primary bidirectional conduit linking the enteric nervous system (ENS) to the CNS [54]. The VN facilitates communication with gut microbiota within the ENS and relays crucial signals to the CNS, where these inputs are interpreted to elicit appropriate physiological responses. For instance, signals associated with gut dysbiosis can prompt the CNS to initiate neuroinflammatory processes, rendering the VN a critical gateway for microbial influence on brain function. Supporting this, Sciopi et al. [55] demonstrated that the VN plays a key regulatory role in mediating the effects of the gut microbiota on brain function and behavior. In their study, healthy mice receiving fecal microbiota transplantation (FMT) from donors subjected to chronic unpredictable mild stress (CUMS) developed depressive-like behaviors and disruptions in serotonergic and dopaminergic signaling, which were dependent on the VN. These alterations, along with hippocampal neurogenesis deficits and neuroinflammation, were completely abrogated by subdiaphragmatic vagotomy, underscoring the VN’s essential role in microbiota-mediated depression. This finding highlights the crucial role of the VN as a major communication route in microbiota-mediated depression. Anatomically, this neural communication is mediated by vagal afferent fibers that interface with the ENS to transmit impulses via the enteric and paravertebral ganglia toward the spinal cord, specifically the T5-L2 and S2-S4 segments, and the brainstem. Within the brainstem, these signals are primarily integrated into the nucleus tractus solitarius (NTS) and the dorsal motor nucleus of the vagus (DMV) [56]. Subsequently, this sensory information is projected to higher-order brain regions, including the hypothalamic paraventricular nucleus (PVN), the striatum, and the amygdala, all of which serve as critical hubs that orchestrate emotional processing and physiological stress responses [57].

At the neuroendocrine level, the HPA axis serves as the body’s primary stress response system and represents another crucial pathway in the MGB axis. In response to stressors, the hypothalamus releases corticotropin-releasing hormone (CRH) and arginine vasopressin, which activate the HPA axis cascade leading to adrenocorticotropic hormone (ACTH) secretion from the anterior pituitary and ultimately glucocorticoid production from the adrenal cortex, such as cortisol in humans and corticosterone in mice [58]. Under physiological conditions, this system is tightly regulated by negative feedback mechanisms. However, gut microbiota dysbiosis can disrupt this delicate balance through several mechanisms, such as the alteration of glucocorticoid receptor sensitivity, modulation of CRH neuronal activity, and induction of inflammation. The resultant hyperactivity of the HPA axis promotes excessive secretion of glucocorticoid, ultimately contributing to depression [59]. Additionally, elevated glucocorticoid may further impair intestinal barrier function and trigger the release of pro-inflammatory cytokines such as IL-1β, IL-6, and TNF-α, thereby forming a vicious cycle that sustains systemic inflammation and neuroinflammation, which is implicated in the pathogenesis of depression [60]. Human studies have identified that patients with depression exhibit gut microbiota dysbiosis alongside the hyperactivity of the HPA axis and elevated cortisol levels [61]. In parallel, animal research using chronic corticosterone-induced mouse models of depression showed that gut dysbiosis triggers ceramide accumulation, which activates the HPA axis, increases corticosterone release, thereby exacerbating depressive-like behaviors [62]. This represents an important pathological mechanism in the development of depression within the MGB axis. Importantly, inflammatory signals can reinforce HPA activation via vagal afferent feedback [63], thereby amplifying stress responses and exacerbating depressive-like behaviors.

**Figure 1:**
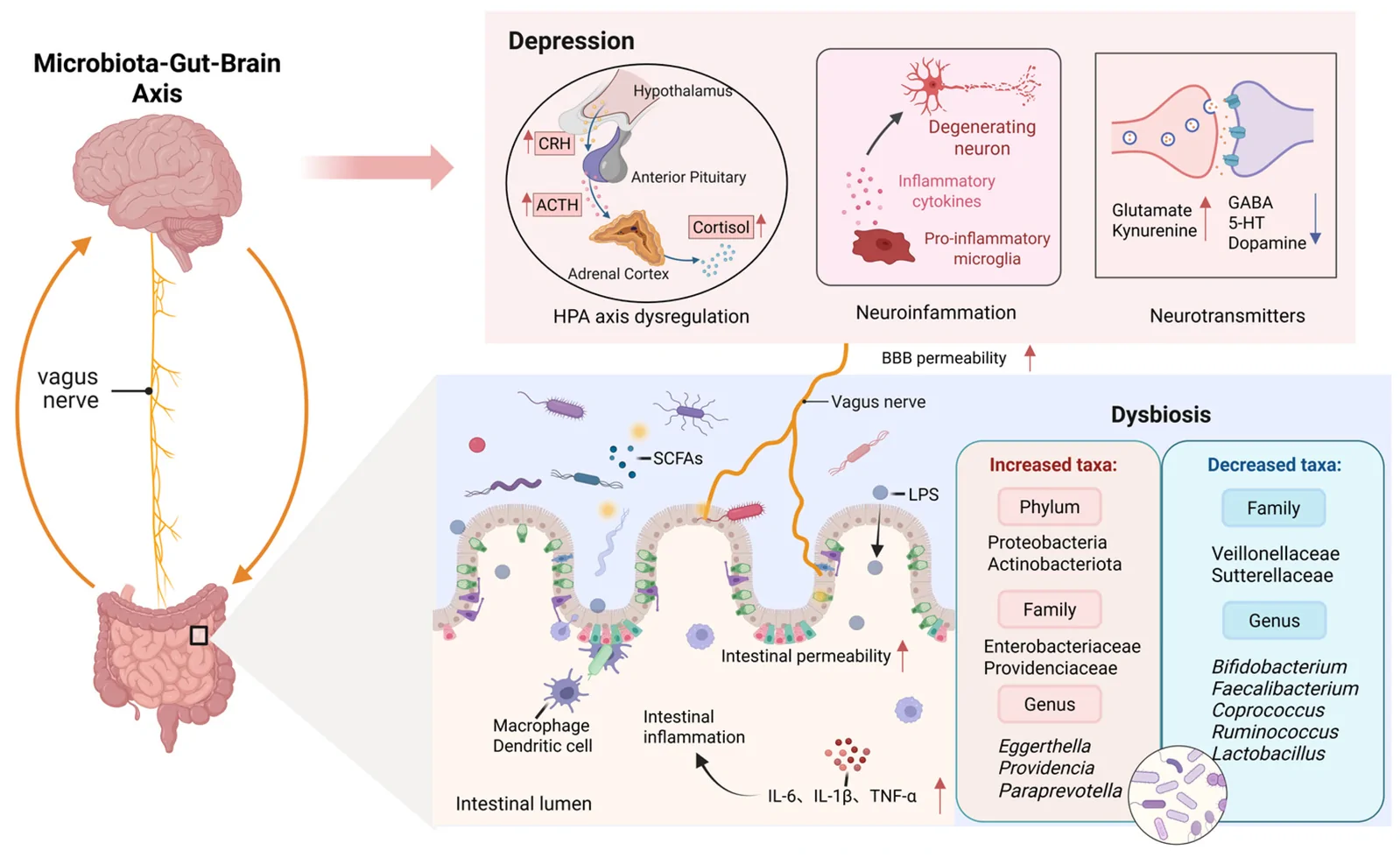
Potential mechanisms of gut microbiota dysbiosis in depression through the MGB axis. Gut microbiota dysbiosis contributes to depression by impairing the integrity of both the intestinal and the BBB, thereby promoting the transmission of harmful signals to the brain. IL-6, interleukin-6; IL-1β, interleukin-1β; TNF-α, tumor necrosis factor-alpha; LPS, lipopolysaccharide; SCFAs, short-chain fatty acids; BBB, blood–brain barrier; HPA, hypothalamic–pituitary–adrenal; CRH, corticotropin-releasing hormone; ACTH, adrenocorticotropic hormone; GABA, gamma-aminobutyric acid; 5-HT, 5-hydroxytryptamine; ↑: increase; ↓: decrease. *Created in BioRender, Xin Kuang. https://BioRender.com/3k2f1co (accessed on 7 August 2026).*

Beyond these primary pathways, gut microbiota influence depression pathogenesis through additional mechanisms involving neurotransmitter systems and tryptophan (Trp) metabolism. Certain microbial species can directly produce or modulate the production of key neurotransmitters including gamma-aminobutyric acid (GABA), dopamine, and 5-HT, which play crucial roles in mood regulation [3]. Moreover, gut dysbiosis shifts Trp metabolism toward the kynurenine pathway. By elevating kynurenine levels, which in turn increases glutamate, it alters the balance between neuroprotective and neurotoxic metabolites, and may thereby adversely affect neuronal function and survival [64]. Collectively, the gut microbiota contributes to the pathogenesis of depression through multiple interconnected pathways, primarily involving immune system activation, VN signaling, and dysregulation of the HPA axis, alongside alterations in neurotransmitter dynamics and Trp metabolism (Figure 1). These pathways function synergistically and are critical in mediating bidirectional communication between the gut and the brain, as depression is a complex brain disorder involving a broad range of pathological mechanisms that warrant further investigation.

## 4. Exercise Modulates Gut Ecosystem

**Figure 2:**
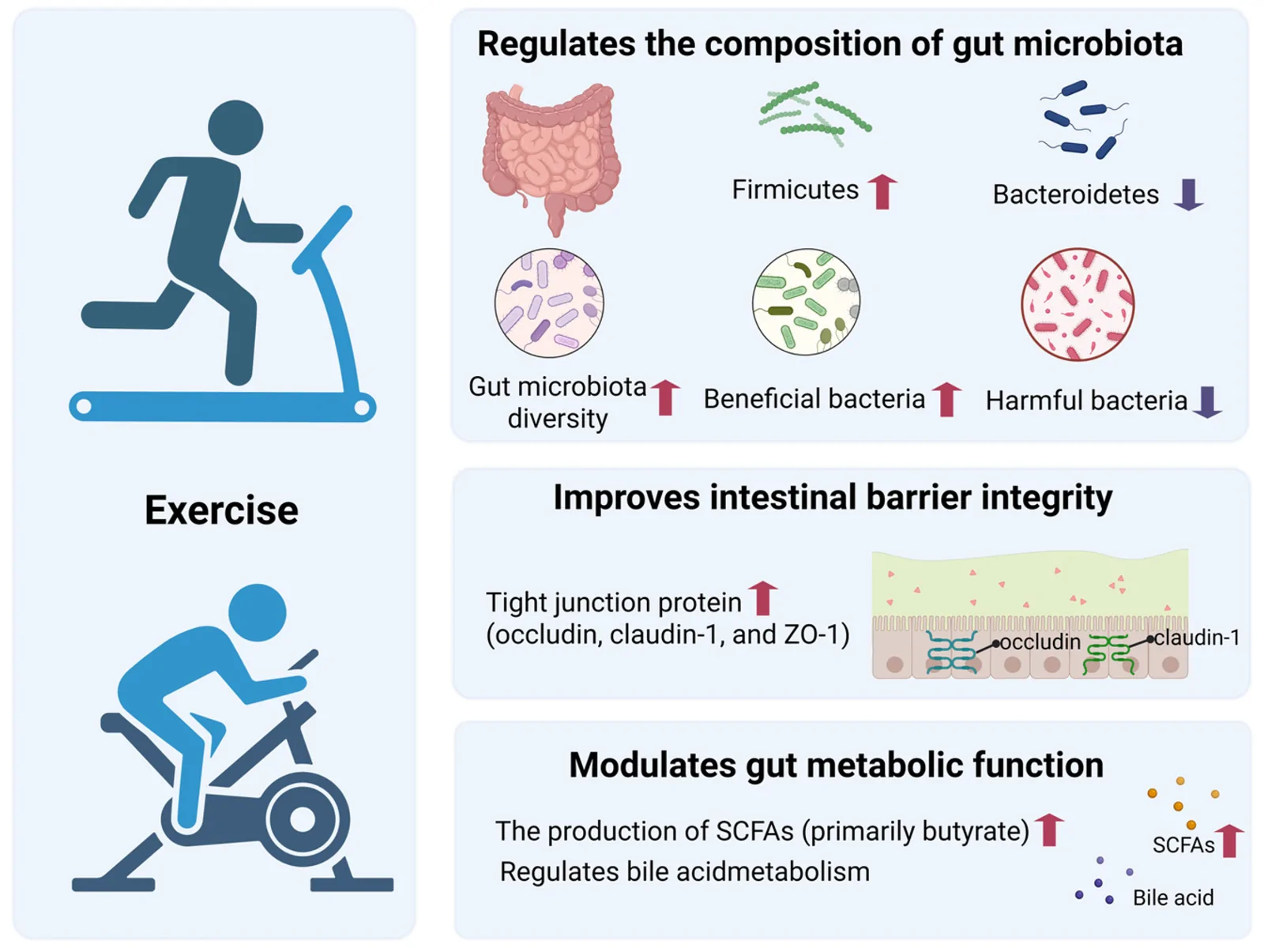
An overview of exercise-associated changes in the gut ecosystem. Exercise influences the gut ecosystem through its effects on microbial diversity, intestinal barrier integrity, and functional metabolism. ZO-1, zonula occludens-1; SCFAs, short-chain fatty acids; ↑: increase; ↓: decrease. *Created in BioRender, Xin Kuang. https://BioRender.com/4zeonfb (accessed on 7 August 2026).*

Exercise is characterized as a planned, structured, and repetitive form of physical activity designed to augment physiological resilience, with aerobic and anaerobic modalities representing the primary classifications. Among these, aerobic exercise has been the most extensively investigated, encompassing common activities such as cycling, running, swimming, and walking. Accumulating evidence indicates that regular aerobic exercise can increase gut microbial diversity, enhance intestinal barrier integrity, and improve functional metabolism in both human and mouse models [65,66], but these responses are not uniform across species or protocols. Their interpretation depends on exercise intensity and duration, dietary exposure, biological sex, baseline microbiota, and host metabolic status. The following sections therefore distinguish preclinical findings from human observations and examine the effects of exercise on the gut ecosystem within the specific conditions under which they were observed (Figure 2).

### 4.1. Exercise Regulates Gut Microbiota Composition

Available human evidence indicates that exercise and habitual physical activity are associated with differences in gut microbial composition and diversity, although the direction and consistency of these differences vary across populations and study designs. Observational comparisons between athletes and sedentary individuals have reported distinct microbial profiles, including greater microbial diversity and enrichment of some taxa with potentially beneficial metabolic functions [67,68]. A compelling example comes from professional rugby players whose gut microbiomes contained 22 distinct microbial phyla, which was exactly double the 11 phyla observed in low-BMI controls. This study further highlighted that the athlete group possessed a higher proportion of Firmicutes and a lower relative abundance of Bacteroidetes compared to high-BMI controls [68]. Because this was a cross-sectional comparison, the observed microbial differences cannot be attributed exclusively to exercise. The athletes also differed substantially from the control groups in dietary intake, particularly total energy and protein consumption, and microbial diversity was positively associated with both protein intake and creatine kinase levels [68]. Therefore, microbial characteristics identified in athlete cohorts should be described as exercise-associated rather than exercise-specific unless dietary exposure has been adequately controlled. Preclinical studies have also identified exercise-associated changes in broad taxonomic composition. Kang et al. [69] reported that wheel-running mice exhibited an elevated abundance of Firmicutes and a reduced abundance of Bacteroidetes, concomitant with enhanced overall microbial diversity compared to sedentary controls. Similarly, Vazquez-Medina et al. [70] demonstrated that a 42-day exercise regimen significantly elevated the F/B ratio in mouse models. Such changes in Firmicutes, Bacteroidetes, and the F/B ratio are not consistently observed across studies and should not be regarded as a universal signature of exercise. Nor should rodent taxonomic patterns be directly extrapolated to humans, given differences in host physiology, microbial ecology, exercise protocols, and baseline microbiota.

Beyond overall diversity, exercise has been associated with selective shifts in the gut ecosystem, characterized by greater abundance of potentially beneficial bacteria and lower abundance of potentially pathogenic species. In rodents, Queipo-Ortuño et al. [71] observed that moderate-intensity exercise in rodents preferentially enhanced the proliferation of beneficial microbes, including Lactobacillus, Bifidobacterium, and the *Blautia coccoides-Eubacterium rectale* cluster, while simultaneously reducing populations of harmful bacteria such as Enterococcus spp. Similarly, Campbell et al. [72] observed enrichment of genera with putative anti-inflammatory properties, including *Faecalibacterium prausnitzii*, Clostridi-um spp., and Allobaculum spp., after voluntary wheel running. Notably, despite the different exercise modalities employed, forced versus voluntary, both interventions converged in their capacity to enrich beneficial bacterial taxa. Human intervention findings are more heterogeneous. A six-week moderate-intensity aerobic intervention was associated with an increased abundance of *Akkermansia muciniphila* and a reduction in some Gram-negative taxa with inflammatory potential [73]. Notably, an elevated abundance of beneficial bacteria such as *Akkermansia muciniphila* has been associated with the alleviation of depressive symptoms [40]. Furthermore, Wang et al. [74] reported increases in the relative abundances of Coprococcus, Blautia, and Dorea following moderate-intensity exercise. Although the functional roles of these genera may vary across species and host conditions, several members have been associated with potentially favorable metabolic and immunoregulatory properties [75,76].

Biological sex may represent an additional source of heterogeneity. Sex-related differences in gut microbial composition have been reported in both humans and mice, and experimental evidence indicates that sex, diet, and exercise can interact in shaping microbial community dynamics [77]. Nevertheless, many exercise-microbiota studies use only male animals or lack adequately powered sex-stratified analyses. Current findings therefore cannot be generalized across sexes without direct comparison.

### 4.2. Exercise Influences Intestinal Barrier Integrity

The intestinal barrier serves as a critical defense system that protects the host from bacterial invasion and exposure to microbial-derived toxic components. Proper intestinal barrier function, maintained by tight junction proteins including occludin, claudin-1, and zonula occludens-1 (ZO-1), is essential for immune homeostasis and brain health. In the context of depression, microbial dysbiosis frequently compromises this epithelial protection, thereby precipitating intestinal barrier dysfunction [78]. The resulting increase in intestinal permeability facilitates the infiltration of harmful substances into the systemic circulation, a pathological state often described as “leaky gut” that triggers widespread inflammatory responses [79]. Preclinical evidence suggests that regular exercise may support intestinal barrier integrity through microbial and epithelial adaptations. Exercise has been associated with increased microbial diversity and enrichment of butyrate-producing taxa, including some Clostridium and Bacteroides species, which may support mucosal repair and epithelial function [80,81]. These microbial changes are accompanied by the upregulation of tight junction proteins, occludin and claudin-1, within the intestinal epithelium, an effect that curbs permeability and limits the systemic translocation of lipopolysaccharides (LPS) and downstream pro-inflammatory cascades [6]. Wang et al. reported that after 12 weeks of treadmill training in overweight mouse models, colonic ZO-1 and occludin expression levels were significantly increased, effectively preventing gut dysbiosis and associated intestinal pathologies [82]. These findings support a potential pathway linking exercise, the gut microbiota, and intestinal barrier function in rodents but do not establish an equivalent mechanism in humans.

Human intervention studies provide more limited evidence. In a randomized controlled trial, an eight-week moderate-intensity running intervention significantly improved intestinal permeability as evidenced by reduced serum lipopolysaccharide-binding protein levels [83]. Pasini et al. also reported that a combined endurance, strength, and flexibility program was associated with changes in microbial composition, improved barrier-related measures, and reduced systemic inflammatory markers in a human cohort [84]. However, it remains uncertain whether these outcomes resulted from the combination of exercise modalities, a greater total training volume, or concurrent changes in dietary and other lifestyle factors.

The effects of exercise on the intestinal barrier also appear to depend on exercise dose. Prolonged or high-intensity exercise has been associated with increased intestinal permeability and elevated intestinal fatty acid-binding protein, a marker of enterocyte injury [85]. Furthermore, overtraining exacerbates intestinal inflammation and compromises epithelial integrity. For instance, in a study employing an overtraining protocol, male mice were subjected to exhaustive treadmill running consisting of two 90-min sessions per day, five days per week, for six consecutive weeks. Compared with sedentary controls, the overtraining group exhibited significantly elevated colonic levels of pro-inflammatory cytokines, including TNF-α and IL-6, reduced expression of tight junction proteins, including occludin and ZO-1, and increased intestinal permeability [86]. These findings demonstrate that exercise-related barrier responses are not uniformly beneficial and may shift toward epithelial injury when total load exceeds recovery capacity. Because only male animals were studied, the exercise threshold associated with barrier disruption in females cannot be inferred from this experiment.

### 4.3. Exercise Modulates Gut Metabolic Function

Preclinical and human studies have both reported associations between exercise and microbial metabolic function, particularly SCFA-related pathways, although the strength and interpretation of the evidence differ. In mice, voluntary wheel running has been associated with changes in microbial composition, immunity, and SCFA-related metabolism [87]. These experimental findings support the capacity of exercise to modify microbial metabolic activity under controlled animal conditions. Human evidence is derived largely from observational comparisons and a smaller number of interventions. Professional athletes have exhibited microbial communities with greater representation of metabolic pathways related to SCFA production than sedentary participants [88,89].

Although the precise mechanisms driving exercise-induced SCFA elevation remain under investigation, proposed explanations include alterations in colonic oxygen availability, luminal pH, gastrointestinal transit, and endogenous metabolites such as lactate, which may modify the intestinal environment and influence the fermentation of dietary substrates, particularly fermentable carbohydrates and fiber [90]. SCFAs may support intestinal barrier and immune homeostasis by regulating tight junction proteins, inflammatory cytokines, and immune activity [91]. Their fecal concentrations, however, reflect the combined effects of microbial production, intestinal absorption, host utilization, and excretion. Diet is therefore a major consideration because the availability of fermentable carbohydrates and fiber directly affects microbial SCFA production and may change concurrently with physical activity. This may partly explain the findings of Magzal et al. [52], who observed higher fecal SCFA concentrations in less active older adults, whereas more active participants showed greater abundances of taxa associated with gut health. Controlled evidence further suggests that diet and exercise may exert both independent and interactive effects on gut microbial networks [92].

Among the various SCFAs, butyrate appears particularly responsive to exercise. As the primary energy substrate for colonic epithelial cells, butyrate exerts critical regulatory effects on intestinal homeostasis [93]. A rodent study employing a five-week voluntary wheel-running regimen demonstrated significant enrichment of butyrate-producing bacterial taxa, including Bacteroidales, Clostridiaceae, Lachnospiraceae, Ruminococcaceae, and Clostridiales [94]. Subsequently, a study employing an eight-week treadmill protocol in C57BL/6 mice confirmed an increase in total butyrate production [95]. Human evidence remains more limited and largely associative, with endurance athletes showing greater microbial diversity and butyrate-producing capacity than sedentary controls [89]. These differences cannot be attributed solely to exercise because athletic populations often differ in dietary intake and other lifestyle characteristics. Butyrate has been shown to epigenetically modulate the expression of genes encoding essential tight junction proteins, including ZO-1, occludin, and claudin-1, thereby reinforcing intestinal barrier integrity [96,97,98]. Beyond SCFA metabolism, animal studies have also reported microbiota-dependent alterations in bile acid metabolism [99]. In a fecal microbiota transplantation experiment, transfer of microbiota from exercised mice to germ-free recipients increased skeletal muscle AMP-activated protein kinase (AMPK) and insulin-like growth factor-1 (IGF-1) levels and was accompanied by greater plasma bile acid deconjugation. These findings indicate that exercise-associated microbial changes may be linked to host metabolic responses under controlled animal conditions. Comparable microbiota-mediated effects have not been established in humans, where the available evidence remains largely associative. The direction and magnitude of these responses may also vary with exercise modality, intensity, duration, and dietary context.

## 5. Exercise Alleviates Depression Through the Microbiota–Gut–Brain Axis

**Figure 3:**
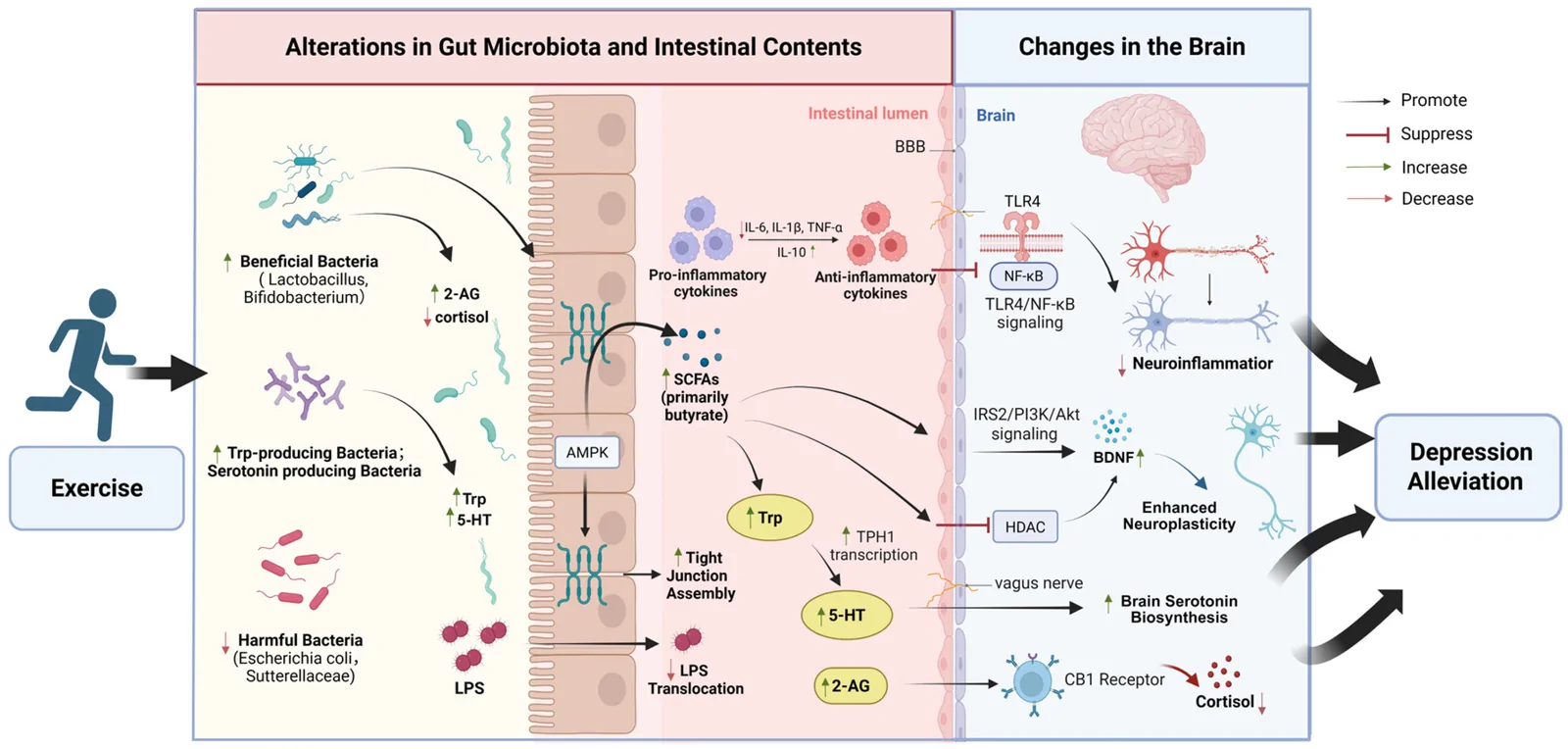
Potential pathways linking exercise-mediated modulation of the MGB axis to depression-related outcomes. Exercise-associated changes in the gut microbiota and microbial metabolites may be related to reduced inflammatory signaling, altered BDNF expression, serotonergic regulation, and modulation of HPA axis activity, which may collectively contribute to improvements in depressive symptoms. Trp, tryptophan; LPS, lipopolysaccharide; AMPK, AMP-activated protein kinase; 5-HT, 5-hydroxytryptamine; 2-AG, 2-arachidonoylglycerol; IL-6, interleukin-6; IL-1β, interleukin-1β; TNF-α, tumor necrosis factor-α; IL-10, interleukin-10; SCFAs, short-chain fatty acids; BBB, blood–brain barrier; TPH1, tryptophan hydroxylase-1; TLR4, Toll-like receptor 4; NF-κB, nuclear factor kappa B; IRS2, insulin receptor substrate 2; PI3K, phosphatidylinositol 3-kinase; Akt, protein kinase B; HDAC, histone deacetylase; BDNF, brain-derived neurotrophic factor; CB1, the type-1 cannabinoid receptor; ↑: increase; ↓: decrease. *Created in BioRender, Xin Kuang. https://BioRender.com/9g8s95q (accessed on 7 August 2026).*

Regular physical exercise has been associated with reduced depressive symptoms and a lower risk of depression [100,101]. However, responses along the MGB axis are unlikely to be uniform or to increase linearly with exercise intensity. Moderate-intensity exercise is frequently associated with changes in microbial composition, intestinal barrier function, microbial metabolism, and inflammatory status, whereas prolonged high-intensity exercise or overtraining may adversely affect intestinal permeability and inflammatory signaling [85,86]. The pathways considered below include neuroinflammatory signaling, BDNF-related neuroplasticity, serotonergic regulation, and HPA axis function (Figure 3). Most depression-related microbiome studies have examined a single exercise protocol, and direct comparisons that vary exercise intensity while simultaneously assessing microbial composition or metabolites and depressive outcomes are currently lacking. Accordingly, the evidence is interpreted in relation to exercise intensity, duration, total workload, recovery, dietary exposure, biological sex, and host characteristics. Controlled animal studies are used to assess mechanistic plausibility, whereas human findings are treated primarily as clinical associations unless mediation has been directly tested.

### 5.1. Exercise Regulates Depression via Inflammation Regulation

Preclinical evidence indicates that exercise-associated changes in the gut environment can occur alongside changes in systemic and central inflammatory activity. In an Alzheimer’s disease mouse model, 12 weeks of treadmill exercise increased microbial diversity and improved indices of intestinal barrier function, accompanied by reduced LPS translocation, systemic inflammatory mediators, microglial activation, and neuroinflammation [102]. Because this study did not use a depression model, its findings support a potential pathway involving the gut microbiota, intestinal barrier, and inflammatory signaling rather than a direct antidepressant mechanism. Specifically, the taxonomic composition and metabolic activity of the gut microbiota plausibly influence inflammatory cytokine secretion. By remodeling gut structure and barrier function, exercise may shift the immune profile from a pro-inflammatory to an anti-inflammatory state, a hypothesis supported primarily by rodent data. For instance, exercise has been shown to increase the abundance of putative probiotic strains with potential antidepressant properties, such as Lactobacillus and Bifidobacterium [103]. These microbes ferment dietary fibers to produce lactate, which has been proposed to activate intestinal GPR81 receptors [104], potentially suppressing the release of pro-inflammatory cytokines (IL-6, TNF-α, IL-1β) while concomitantly upregulating IL-10 [105]. Moreover, exercise interventions have also been associated with lower abundances of potentially pro-inflammatory bacteria such as *Escherichia coli* [106]. Reduced *E. coli* abundance may limit microbial LPS availability and attenuate downstream TLR4/nuclear factor kappa B (NF-κB) signaling, a pathway implicated in neuroinflammation and depression-related behavioral abnormalities [107], although the specific contribution of this pathway to depression-related outcomes has not been established in humans. More directly relevant evidence comes from a depression-related rodent model, in which four weeks of swimming prevented the increase in *E. coli* and the decrease in Lactobacillus. These microbial changes were accompanied by lower hippocampal levels of pro-inflammatory cytokines and improved depressive-like behaviors. The authors hypothesized that these exercise-associated adaptations may support intestinal immune homeostasis and attenuate neuroinflammation, thereby potentially contributing to the observed improvements in depressive-like behavior [108]. Notably, these inflammation-related responses may vary with exercise dose. Compared with low-intensity running, high-intensity running has been associated with transient increases in intestinal permeability, neutrophil recruitment, and intestinal IL-1β and IL-6 expression in mice [109]. These findings suggest that the barrier and inflammatory responses observed with regular or moderate exercise may not extend uniformly to higher exercise intensities. Whether such changes subsequently influence microbiota-related inflammatory signaling or depression-related outcomes has not been directly established.

The anti-inflammatory properties of SCFAs have been extensively documented, particularly their capacity to modulate pro-inflammatory cytokines, inhibit NF-κB signaling, and support microglial homeostasis [110,111]. In human studies, higher SCFA levels have been associated with less severe depressive symptoms, although these associations do not establish directionality or mediation [21]. A six-week exercise intervention in older adults was accompanied by increased abundances of Bifidobacterium and Faecalibacterium, greater butyrate production, and lower circulating TNF-α and IL-6 concentrations [112]. Because these outcomes changed concurrently, the study does not demonstrate that butyrate mediated the reduction in inflammatory markers. Independent experimental evidence nevertheless supports several anti-inflammatory actions of butyrate. Butyrate can regulate neutrophil migration and reduce cytokine-induced expression of vascular cell adhesion molecule-1 (VCAM-1) [113]. It can also activate AMPK in intestinal epithelial cells and promote tight junction assembly, thereby supporting barrier integrity [114]. In models of LPS-induced neuroinflammation, greater butyrate availability has been associated with reduced inflammatory signaling and improved behavioral outcomes [115]. These findings make butyrate-mediated immune regulation a biologically plausible component of exercise-associated responses, whereas its contribution to antidepressant effects in humans remains inferential.

The VN may represent an additional pathway linking exercise-associated autonomic adaptations with intestinal and inflammatory signaling. Regular training enhances autonomic function and improves vagal tone, facilitating bidirectional signaling between the gut and the CNS [116]. Activation of the VN initiates the cholinergic anti-inflammatory pathway, which stimulates T cells to release acetylcholine. This neurotransmitter subsequently binds to α7 nicotinic acetylcholine receptors (α7nAChR) on macrophages and other immune cells, thereby suppressing NF-κB signaling and reducing cytokine storm potential [117]. Importantly, studies have shown that VN stimulation effectively counteracts endotoxin-induced inflammation. In contrast, vagotomy without electrical stimulation resulted in increased levels of TNF-α [118]. Vagal cholinergic signaling may therefore connect exercise-related autonomic changes with inflammatory regulation along the MGB axis, but its relationship with microbial alterations and depressive outcomes has not been directly demonstrated in patients with depression.

### 5.2. Exercise Regulates Depression Through BDNF Modulation

BDNF is a widely expressed neurotrophin involved in neuronal survival, differentiation, and synaptic plasticity across multiple brain regions. Reduced BDNF signaling and impaired hippocampal neurogenesis have been implicated in the pathophysiology of depression. Alterations in gut microbial composition have also been associated with these neurotrophic changes, although the supporting mechanistic evidence is derived mainly from preclinical studies [119]. Bifidobacterium and Lactobacillus have been associated with circulating or central BDNF levels, hippocampal neurogenesis, and the severity of depressive phenotypes [120,121], suggesting a potential relationship between selected microbial taxa, neurotrophic regulation, and depression-related outcomes. Exercise-associated changes in these taxa have also been reported in animal models. Following 10 weeks of voluntary wheel running, increases in Bifidobacterium and Lactobacillus occurred alongside higher BDNF protein expression in the hippocampus and prefrontal cortex and enhanced synaptogenesis [122]. These concurrent changes are consistent with a relationship among exercise, microbial composition, and neurotrophic signaling, but do not demonstrate that the microbial alterations mediated the increase in BDNF. More direct evidence linking exercise, microbiota disruption, and neurogenesis comes from adult male rats, in which voluntary running attenuated reductions in adult hippocampal neurogenesis and associated behavioral impairments caused by microbiota disruption [123]. Because this study included only males, the extent to which comparable responses occur in females cannot be determined. Complementing this evidence, a study that included both male and female mice found that voluntary wheel running produced sex-specific changes in behavior, cecal microbial composition, and intestinal and brain nutritional biomarkers [124]. These results indicate that exercise-associated microbiota and brain-related responses may differ between females and males. Furthermore, exercise has also been associated with greater abundance of *Akkermansia muciniphila* [125], whereas supplementation with this species attenuated stress-related depressive-like behaviors and was accompanied by changes in microbial composition, neurotransmitters, hormones, and BDNF expression in a separate experimental model [126]. The overlap between these findings suggests a potential microbiota–BDNF pathway but does not establish that exercise acts through the same mechanism. BDNF expression has additionally been reported to correlate negatively with potentially pathogenic families, including Helicobacteriaceae, Enterobacteriaceae, and Sutterellaceae [127], while exercise interventions have been associated with lower abundances of some of these taxa [128]. Overall, current animal evidence suggests that microbial composition may contribute to exercise-associated neurotrophic regulation, although the relevant taxa, immune responses, and behavioral outcomes may differ by sex.

Microbial metabolites, particularly SCFAs, serve as critical stimulators of growth factors that support hippocampal function. Specifically, SCFAs can trigger AMPK phosphorylation in muscle cells, which subsequently activates downstream targets such as peroxisome proliferator-activated receptor gamma coactivator-1α (PGC-1α) and p38 mitogen-activated protein kinases (MAPK), both of which are essential for BDNF biosynthesis [129]. Importantly, higher SCFA availability has also been associated with increased BDNF expression and improved depressive-like behaviors [130]. Given that exercise elevates SCFA levels [84,85], it is reasonable to speculate that exercise may alleviate depression through an SCFA-mediated upregulation of BDNF. However, further human and animal studies are required to validate this hypothesis. Furthermore, butyrate is a metabolite primarily produced by gut bacteria such as Faecalibacterium, which is a major component of the butyrate-producing microbial community. Butyrate has been associated with the maintenance of BDNF levels and the support of hippocampal neurogenesis, which may contribute to the alleviation of depressive behaviors [131]. Exercise-associated increases in Faecalibacterium and endogenous butyrate levels have also been reported [89,93]. These findings should be interpreted in relation to dietary intake because the availability of fermentable substrates directly influences microbial butyrate production and may change concurrently with physical activity. In C57BL/6 mice exposed to a high-fat, high-cholesterol diet, fecal microbiota transplantation from exercised donors was accompanied by increased hippocampal BDNF, PSD95, and synaptophysin expression, altered IRS2/PI3K/Akt signaling, and reduced HDAC2 and HDAC3 levels [132]. These findings demonstrate the transferability of an exercise-associated microbial phenotype under a defined dietary challenge, but they neither isolate butyrate as the responsible metabolite nor establish an antidepressant mechanism in humans. In conclusion, the available evidence indicates that changes in the gut microbiota and microbial metabolism may contribute to BDNF-related responses to exercise. The key unresolved question is whether exercise-associated changes in butyrate or other microbial metabolites account for measurable BDNF and depressive symptom responses in humans after dietary exposure has been adequately controlled.

### 5.3. Exercise Modulates Depression via 5-HT Synthesis

Serotonergic signaling represents one route through which exercise-associated changes in the MGB axis may relate to depressive outcomes. As a fundamental neurotransmitter in mood regulation, 5-HT deficiency is strongly implicated in the pathogenesis of depressive disorders [133]. Notably, more than 90% of total body 5-HT is synthesized peripherally within the gastrointestinal tract, a process initiated by the essential amino acid Trp and catalyzed by the rate-limiting enzyme tryptophan hydroxylase (TPH). The TPH1 isoform, which is specifically expressed in enterochromaffin (EC) cells, drives this predominant peripheral production, whereas the TPH2 isoform mediates central synthesis within neurons [134]. The gut microbiota is essential for regulating this peripheral 5-HT homeostasis. Specifically, SCFAs produced by phyla such as Firmicutes and Bacteroidetes have been shown to stimulate TPH1 transcription in colonic EC cells [135]. Within this metabolic landscape, butyrate is of particular significance. Research by Yano et al. [136] demonstrated that spore-forming bacteria, such as Bacillus and Clostridium, enhance intestinal 5-HT synthesis precisely by specifically upregulating TPH1 expression in EC cells via metabolites including butyrate. This intestinal 5-HT may activate 5-HT receptors on the afferent terminals of the VN, which then relay signals to brainstem nuclei [137]. Importantly, exercise can increase the abundance of spore-forming bacteria, an effect that may be relevant to the alleviation of depressive symptoms. Preliminary evidence from an obese mouse model supports this correlation, as exercise increased the abundance of spore-forming bacteria such as Bacillus and Clostridium while concurrently reducing depression-like behaviors, an effect associated with stimulated 5-HT release [138]. In addition, supplementation with *Bifidobacterium* has been shown to elevate hippocampal 5-HT and alleviate depressive behaviors, an effect associated with increased colonic butyrate and subsequent TPH1 expression. Given that exercise can also increase Bifidobacterium abundance [77], it is plausible that exercise may influence 5-HT synthesis and contribute to antidepressant effects through a similar pathway.

Exercise may also be associated with changes in Trp metabolism, although find-ings differ across compartments and protocols. In cross-country endurance athletes, acute moderate-intensity exercise increased the abundances of Romboutsia, Ruminococcaceae UCG-005, and Blautia, whereas Ruminiclostridium 9 decreased; fecal Trp was positively associated with Romboutsia [139,140]. Interestingly, reduced serum Trp levels have been observed in participants following cycling tests and after single bouts of endurance exercise [141,142]. This shift may reflect increased gut utilization of Trp for local 5-HT production, which may be associated with changes in central serotonergic function and mood-related outcomes. Additionally, several bacterial species within the gut microbiota, including *Lactococcus lactis*, *Lactobacillus plantarum*, *Streptococcus thermophilus* [143], have shown the capacity to produce 5-HT under defined in vitro conditions. Exercise has been associated with increased abundances of bacteria with reported 5-HT-producing capacity [144]. These microbial changes may occur alongside alterations in 5-HT availability and depressive symptoms, although their directionality and mediating role have not been established. Taken together, current evidence suggests that exercise-related changes in the gut microbiota may be linked to serotonergic regulation through several potentially convergent routes: (1) enrichment of bacteria that produce metabolites, particularly butyrate, which may stimulate TPH1 expression; (2) increased abundance of bacteria involved in Trp biosynthesis or metabolism; and (3) changes in bacteria with the reported capacity to synthesize 5-HT directly.

### 5.4. Exercise Modulates Depression Through HPA Axis Regulation

Substantial evidence suggests that the modulation of the HPA axis via the MGB axis is a potential pathway relevant to exercise-associated changes in depressive symptoms. This intersystem connection is supported by preclinical studies demonstrating that specific probiotic interventions, particularly those involving Lactobacillus and Bifidobacterium, can effectively suppress stress-induced HPA axis hyperactivity and mitigate depressive-like behaviors [145,146]. Specifically, the administration of Lactobacillus has been shown to reinforce intestinal barrier integrity, thereby attenuating HPA axis reactivity to physical stressors such as partial restraint [147]. While clinical research in humans remains developing, preliminary investigations have identified significant correlations between the abundance of these microbial taxa and systemic cortisol levels. In a randomized controlled trial involving healthy volunteers, supplementation with *Lactobacillus helveticus* R0052 and *Bifidobacterium longum* R0175 was accompanied by reduced psychological distress and lower cortisol levels [148]. This effect coincided with reduced cortisol levels, although the study was not conducted in a clinically depressed population. Importantly, these beneficial microbial populations are frequently enriched following exercise interventions. Evidence from exercise studies currently comes from two biologically distinct settings. Under controlled rodent conditions, voluntary wheel running increased the relative abundances of Bifidobacterium and Lactobacillus, while selected Lactobacillus strains have also been linked to pathogen suppression and intestinal barrier maintenance [74]. In the clinical study by Mahdieh et al. [122], 12 weeks of aerobic exercise in older adults with depression was followed by increases in the same two genera, together with lower cortisol concentrations and reduced depressive symptoms. The human findings therefore describe parallel microbial, endocrine, and clinical responses, rather than a demonstrated sequence in which microbial changes regulate the HPA axis and subsequently improve depression. Moreover, the recurrence of Lactobacillus and Bifidobacterium across the two studies represents overlap only at a broad genus level, because rodent and human gut ecosystems differ markedly in their underlying taxonomic structure and ecological function. Evidence regarding other exercise-responsive microorganisms and their relationship with HPA axis activity in depression remains limited.

The endocannabinoid (eCB) system plays a critical role in regulating the activation of the HPA axis. Primary eCB ligands such as anandamide and 2-arachidonoylglycerol (2-AG) activate cannabinoid receptors type-1 (CB1) and type-2 (CB2) to maintain physiological homeostasis [149]. CB1 receptors are highly expressed within the mesolimbic pathway and appear to exert inhibitory control over HPA axis-mediated stress responses, although chronic stress has been shown to disrupt normal CB1 receptor signaling, potentially impairing this inhibitory feedback and contributing to persistent hypothalamic activation [150]. Notably, in animal models of chronic stress, gut dysbiosis and reduced SCFA availability have occurred alongside impaired eCB signaling, elevated corticosterone, and reduced hippocampal neurogenesis [151]. Recent studies indicate that supplementation with selected Lactobacillus strains or colonization with *Akkermansia muciniphila* was accompanied by changes in eCB-related signaling, higher intestinal 2-AG levels, improved barrier integrity, and reduced depressive-like behaviors [151,152]. Furthermore, SCFAs have been reported to influence the expression of genes involved in HPA axis regulation through epigenetic mechanisms [153]. Exercise has also been associated with increased abundances of selected taxa, including Lactobacillus and *Akkermansia muciniphila*, together with changes in SCFA production [122,154]. These responses may contribute to an intestinal environment associated with eCB signaling and HPA axis regulation. Based on the evidence reviewed above, we hypothesize that exercise-associated changes along the MGB axis may be linked to antidepressant outcomes through interconnected microbial, metabolic, and neuroendocrine pathways. These pathways may involve alterations in microbial composition, SCFA availability, and eCB-related signaling, which may occur alongside reduced HPA axis hyperactivity. However, most evidence concerning interactions among the gut microbiota, eCB system, and HPA axis is derived from animal or other preclinical models. Human evidence remains limited and predominantly associative, and it has not been established whether microbial and eCB-related changes mediate HPA axis responses or occur in parallel with other physiological adaptations to exercise.

## 6. Limitations of the Current Evidence

Despite the comprehensive synthesis provided in this review, several important limitations should be acknowledged when interpreting the available evidence.

Much of the mechanistic evidence regarding the interactions among exercise, gut microbiota, and depression derives from animal models or preclinical studies. While these studies offer valuable insights into potential pathways, including the regulation of intestinal barrier integrity, SCFA production, BDNF expression, and HPA axis activity, direct evidence in humans remains scarce. Most available human data are correlational in nature, and it remains unclear whether the observed microbial changes directly drive the antidepressant effects of exercise or merely accompany other physiological adaptations. Furthermore, the translational relevance of the proposed pathways to clinical populations remains uncertain, as findings from animal models may not fully recapitulate the complex pathophysiology of human depression.

The heterogeneity of exercise protocols across studies, including variations in type, frequency, intensity, duration, and modality, complicates cross-study comparisons and limits the generalizability of findings. The lack of standardized exercise prescriptions makes it difficult to determine the optimal regimen for maximizing antidepressant efficacy. In addition, substantial interindividual variability in gut microbial composition, driven by genetic background, dietary habits, medication use, and lifestyle factors, poses a major challenge to the identification of a universal microbial signature associated with exercise response and limits the applicability of current evidence to heterogeneous patient subgroups.

The difficulty of establishing causal relationships between specific microbial changes and depressive outcomes remains a critical issue. Most studies are cross-sectional and cannot distinguish between correlation and causation. Furthermore, potential confounding factors, such as diet, physical activity levels outside the intervention, sleep quality, and concomitant medication, are frequently underreported or insufficiently controlled. The temporal dynamics of microbial changes during exercise intervention are also poorly characterized, as few studies have employed longitudinal designs with repeated measurements across multiple time points. Research has largely focused on a narrow range of bacterial genera, such as Lactobacillus and Bifidobacterium, while the involvement of other microbial taxa and their functional contributions to the MGB axis remains largely unexplored.

Collectively, these limitations highlight the need for well-controlled longitudinal intervention studies with standardized exercise protocols, comprehensive confounder adjustment, and repeated measurements of microbial, metabolic, and clinical outcomes to establish causality and inform the development of personalized exercise-based interventions for depression.

## 7. Conclusions and Future Directions

The dysfunction of the MGB axis presents a central challenge in depression, contributing to its complex pathophysiology through dysregulations across multiple physiological systems. The evidence synthesized in this review underscores that exercise serves as a potent intervention for alleviating depressive symptoms primarily by remodeling the gut microbial ecosystem. We have comprehensively demonstrated that the antidepressant effects of exercise are deeply rooted in the modulation of the gut microbiota, which subsequently influences immune, neural, endocrine, and metabolic pathways. Specifically, exercise promotes a favorable taxonomic shift characterized by an enriched abundance of beneficial bacteria and SCFAs alongside the suppression of pathogenic taxa. This restoration of microbial equilibrium facilitates an anti-inflammatory environment that mitigates neuroinflammation and preserves neuronal health. Furthermore, exercise-mediated microbial changes stimulate the expression of BDNF and promote 5-HT synthesis. Finally, the normalization of the HPA axis is achieved through the integrated action of microbial metabolites and the recovery of the eCB system, all of which collectively establish the biological foundation for exercise as a therapeutic strategy for depression via the MGB axis.

Despite significant advancements in elucidating the link between exercise and the gut microbiota, several critical gaps remain to be addressed. Current research has largely focused on specific microbial taxa and isolated metabolic pathways, yet the gut microbiota constitutes a highly complex and dynamic ecosystem shaped by host genetics, dietary patterns, environmental exposures, and circadian rhythms. Consequently, limited and static microbial indicators are insufficient to capture the full structural and functional state of the entire microbial community, nor can they adequately elucidate the holistic role of gut microbiota in the development and progression of depression.

Well-designed longitudinal and randomized controlled trials with standardized exercise protocols are urgently needed to establish causal relationships between exercise induced microbial changes and antidepressant outcomes. Such studies should incorporate repeated measurements of microbial composition, functional metabolites, neuroendocrine markers, and clinical endpoints, combined with mediation analyses to determine whether specific microbial shifts directly mediate improvements in depressive symptoms or merely accompany other physiological adaptations. Future research should develop analytical approaches targeting individualized microbiome and metabolome profiles to identify depression-specific biomarkers, thereby providing a foundation for precision interventions. The identification of microbiota derived biomarkers that predict individual responsiveness to exercise would represent a critical step toward personalized medicine. Stratified analyses based on baseline microbial composition, genetic background, and clinical characteristics could help determine which depressed patients are most likely to benefit from exercise induced modulation of the MGB axis.

Furthermore, there is currently no consensus regarding the optimal exercise prescription required to maximize antidepressant outcomes. Various exercise modalities, including endurance training and resistance training, as well as differences in intensity and duration, may lead to distinct changes in gut microbial composition and depressive symptoms. However, comparative studies in this field remain limited. It is therefore imperative to comprehensively evaluate how different forms of exercise influence the microbiota–gut–brain axis to support the development of individualized exercise protocols. The development of tailored exercise protocols that can selectively modulate the MGB axis based on baseline microbial profiles, metabolic status, and individual clinical features holds great promise for optimizing antidepressant efficacy. This requires systematic investigation of dose response relationships between exercise parameters, including type, frequency, intensity, and duration, and specific microbial and neuroendocrine outcomes.

Additionally, although previous research has primarily focused on single intervention approaches such as probiotic supplementation, integrating exercise with other microbiota directed strategies, for instance prebiotics, symbiotics, dietary fiber, or fecal microbiota transplantation, may yield synergistic benefits through multi-target and multi-pathway mechanisms. Such an integrated framework could pave the way for more effective and personalized therapeutic strategies, ultimately transforming the prevention and treatment of depression in the era of molecular medicine. Addressing these research priorities will be essential for moving the field from descriptive associations to mechanism based, individualized therapeutic strategies that can be translated into clinical practice.

## Data Availability

No new data were created or analyzed in this study. Data sharing is not applicable to this article.

## Abbreviations

The following abbreviations are used in this manuscript:

MDD	Major Depressive Disorder
MGB	Microbiota–gut–brain
VN	Vagus nerve
F/B	Firmicutes-to-Bacteroidetes
SCFAs	Short-chain fatty acids
GPCRs	G protein-coupled receptors
HDACs	Histone deacetylases
PPARγ	Peroxisome proliferator-activated receptor γ
FXR	Farnesoid X receptor
TGR5	Takeda G-coupled protein receptor 5
5-HT	5-hydroxytryptamine
LPS	Lipopolysaccharide
TLR4	Toll-like receptor 4
IL-1β	Interleukin-1β
IL-6	Interleukin-6
TNF-α	Tumor necrosis factor-alpha
BBB	Blood–brain barrier
CNS	Central nervous system
ENS	Enteric nervous system
FMT	Fecal microbiota transplantation
CUMS	Chronic unpredictable mild stress
NTS	Nucleus tractus solitarius
DMV	Dorsal motor nucleus of the vagus
PVN	Paraventricular nucleus
CRH	Corticotropin-releasing hormone
ACTH	Adrenocorticotropic hormone
Trp	Tryptophan
GABA	Gamma-aminobutyric acid
AD	Alzheimer’s disease
ZO-1	Zonula occludens-1
AMPK	AMP-activated protein kinase
IGF1	Insulin-like growth factor-1
BDNF	Brain-derived neurotrophic factor
IL-10	Interleukin-10
NF-κB	Nuclear factor kappa B
MCP-1	Monocyte Chemoattractant Protein-1
VCAM-1	Vascular cell adhesion molecule-1
α7nAChR	α7 nicotinic acetylcholine receptors
PGC-1α	Peroxisome proliferator-activated receptor gamma coactivator-1α
MAPK	Mitogen-activated protein kinases
IRS2	Insulin receptor substrate 2
PI3K	Phosphatidylinositol 3-kinase
AKT	Protein kinase B
TPH	Tryptophan hydroxylase
EC	Enterochromaffin
eCB	Endocannabinoid
2-AG	2-arachidonoylglycerol
CB-1	Cannabinoid receptors type-1
CB-2	Cannabinoid receptors type-2
